# Supplementary material for: The Impact of Dietary Sugars and Saturated Fats on Body and Liver Fat in a Healthcare Worker Population
Source: Nutrients. 2025 Apr 11;17(8):1328. doi: 10.3390/nu17081328 (PMC12029709; doi:10.3390/nu17081328)
Supplement: Supplementary file 1 [file nutrients-17-01328-s001.zip › Supplementary_File_S1 DAGEB.pdf]

HABITUAL DIET ( ) R24 ( )

Date: ________________________

**Meal**	**Details**
Breakfast	
Snack	
Lunch	
Snack	
Dinner	

Forgotten Foods

- Coffee, tea, milk, atole
- Juice, flavored water, soft drinks
- Beer, wine, tequila
- Candy, caramel, gum
- Gelatin, ice cream, flan
- Peanuts, nuts, pistachios
- Chips, nachos, popcorn
- Fresh or dried fruits
- Jicama, carrots, cucumbers
- Cereal, bread, tortilla
- Oil, butter, cream
- Dressing, sauce, avocado
- Cheese, yogurt
- Bacon, croutons

Macronutrient Breakdown

**% VET**	**Energy**	**Grams**
Carbohydrates		
Proteins		
Fats		
**Total**		

Next Consultation Goal

____

**Observations**<br>______________________________	**Water Intake**<br>______________________________
**Weekend Changes**<br>______________________________	**Nutritional Diagnosis**<br>______________________________
**Plan**<br>______________________________	

Adherence to Diet

0 10

Adherence to Exercise

0 10
