# Supplementary material for: The Impact of Dietary Sugars and Saturated Fats on Body and Liver Fat in a Healthcare Worker Population
Source: Nutrients. 2025 Apr 11;17(8):1328. doi: 10.3390/nu17081328 (PMC12029709; doi:10.3390/nu17081328)
Supplement: Supplementary file 1 [file nutrients-17-01328-s001.zip › Supplementary_Table_S1 DAGEB.pdf]

Supplementary Table S1. Multivariable logistic regression models on the relationship of dietary intake quartiles with the presence of hepatic steatosis.

	**B coefficient**	**Standard Error**	**OR (95%CI)**	**P value**
Kilocalories Quartile 1	Reference			
Kilocalories Quartile 2	-0.236	0.326	0.790 (0.417-1.497)	0.469
Kilocalories quartile 3	-0.424	0.397	0.655 (0.301-1.424)	0.286
Kilocalories Quartile 4	-0.254	0.578	0.775 (0.250-2.406)	0.660
Carbohydrate quartile 1	Reference			
Carbohydrates quartile 2	0.175	0.329	1.191 (0.625-2.268)	0.595
Carbohydrate quartile 3	0.568	0.309	1.765 (0.963-3.236)	0.066
Carbohydrate quartile 4	0.666	0.370	1.947 (0.942-4.022)	0.072
Quartile protein 1	Reference			
Quartile Protein 2	-0.984	0.322	0.374 (0.199-0.703)	0.002
Quartile Protein 3	-0.425	0.340	0.654 (0.336-1.273)	0.211
Quartile protein 4	-0.225	0.403	0.798 (0.363-1.759)	0.576
Quartile Fat 1	Reference			
Quartile Fat 2	-0.317	0.316	0.728 (0.392-1.352)	0.315
Quartile Fat 3	0.159	0.362	1.172 (0.577-2.381)	0.661
Quartile Fat 4	-0.586	0.341	0.556 (0.285-1.086)	0.086
Saturated Fat Quartile 1	Reference			
Saturated Fat Quartile 2	0.084	0.310	1.088 (0.592-1.999)	0.787
Saturated Fat Quartile 3	0.308	0.361	1.360 (0.671-2.759)	0.394
Saturated Fat Quartile 4	-0.432	0.340	0.649 (0.334-1.264)	0.204
Total sugars quartile 1	Reference			
Total sugars quartile 2	-0.598	0.313	0.550 (0.298-1.014)	0.056
Total sugars quartile 3	-0.013	0.321	0.987 (0.526-1.852)	0.967
Total sugars quartile 4	0.049	0.324	1.051 (0.556-1.984)	0.879
Added sugar quartile 1	Reference			
Added sugar quartile 2	-0.045	0.303	0.956 (0.528-1.732)	0.883
Added sugar quartile 3	0.251	0.307	1.286 (0.704-2.348)	0.414
Added sugar quartile 4	0.013	0.311	1.013 (0.551-1.864)	0.967
Fructose Quartile 1	Reference			
Fructose Quartile 2	0.029	0.300	1.030 (0.572-1.854)	0.922
Fructose Quartile 3	-0.785	0.310	0.456 (0.249-0.838)	0.011
Fructose Quartile 4	-0.185	0.323	0.831 (0.441-1.565)	0.566
Protein (%) quartile 1	Reference			
Protein (%) quartile 2	-0.362	0.314	0.696 (0.376-1.288)	0.249
Protein (%) quartile 3	-0.013	0.321	0.987 (0.526-1.853)	0.967
Protein (%) quartile 4	-0.617	0.328	0.540 (0.283-1.027)	0.060
Carbohydrates (%) quartile 1	Reference			
Carbohydrates (%) quartile 2	0.076	0.319	1.079 (0.577-2.018)	0.812
Carbohydrates (%) quartile 3	0.795	0.315	2.214 (1.193-4.109)	0.012
Carbohydrates (%) quartile 4	0.351	0.320	1.420 (0.759-2.659)	0.273
Fat (%) quartile 1	Reference			
Fat (%) quartile 2	0.123	0.312	1.131 (0.613-2.087)	0.693
Fat (%) quartile 3	0.129	0.308	1.137 (0.622-2.081)	0.676
Fat (%) quartile 4	-0.410	0.315	0.663 (0.358-1.229)	0.192
Total sugar (%) quartile 1	Reference			
Total sugar (%) quartile 2	-0.150	0.310	0.861 (0.469-1.581)	0.629

Total sugar (%) quartile 3	-0.465	0.326	0.628 (0.331-1.189)	0.153
Total sugar (%) quartile 4	0.215	0.309	1.239 (0.676-2.272)	0.488
Saturated Fat (%) Quartile 1	Reference			
Saturated Fat (%) Quartile 2	-0.133	0.317	0.875 (0.470-1.630)	0.674
Saturated Fat (%) Quartile 3	0.176	0.317	1.193 (0.641-2.219)	0.578
Saturated Fat (%) Quartile 4	-0.424	0.328	0.655 (0.344-1.246)	0.197
Added sugar (%) quartile 1	Reference			
Added sugar (%) quartile 2	-0.417	0.325	0.659 (0.348-1.247)	0.200
Added sugar (%) quartile 3	0.152	0.298	1.165 (0.650-2.088)	0.609
Added sugar (%) quartile 4	-0.066	0.311	0.936 (0.509-1.721)	0.833
Fructose (%) quartile 1	Reference			
Fructose (%) quartile 2	-0.029	0.314	0.971 (0.525-1.797)	0.926
Fructose (%) quartile 3	-0.762	0.324	0.467 (0.247-0.880)	0.019
Fructose (%) quartile 4	0.006	0.310	1.006 (0.548-1.845)	0.985

Models adjusted for age, sex, BMI, Waist circumference and total Kcal
